# The effect of glial fibrillary acidic protein expression on neurite outgrowth from retinal explants in a permissive environment

**DOI:** 10.1186/1756-0500-5-693

**Published:** 2012-12-22

**Authors:** Kimberly A Toops, Tracy L Hagemann, Albee Messing, Robert W Nickells

**Affiliations:** 1 Departments of Ophthalmology and Visual Sciences, University of Wisconsin, Madison, WI, 53706, USA; 2 Departments of Comparative Biosciences, University of Wisconsin, Madison, WI, 53706, USA; 3 The Waisman Center, University of Wisconsin, Madison, WI, 53706, USA; 4 Medical Sciences Center, 1300 University Avenue, Madison, WI, 53706-1532, USA

**Keywords:** Glial fibrillary acidic protein, Axon regeneration, Retinal ganglion cell

## Abstract

**Background:**

Increased expression of glial fibrillary acidic protein (GFAP) within macroglia is commonly seen as a hallmark of glial activation after damage within the central nervous system, including the retina. The increased expression of GFAP in glia is also considered part of the pathologically inhibitory environment for regeneration of axons from damaged neurons. Recent studies have raised the possibility that reactive gliosis and increased GFAP cannot automatically be assumed to be negative events for the surrounding neurons and that the context of the reactive gliosis is critical to whether neurons benefit or suffer. We utilized transgenic mice expressing a range of *Gfap* to titrate the amount of GFAP in retinal explants to investigate the relationship between GFAP concentration and the regenerative potential of retinal ganglion cells.

**Findings:**

Explants from *Gfap*^*-/-*^ and *Gfap*^*+/-*^ mice did not have increased neurite outgrowth compared with *Gfap*^*+/+*^ or *Gfap* over-expressing mice as would be expected if GFAP was detrimental to axon regeneration. In fact, *Gfap* over-expressing explants had the most neurite outgrowth when treated with a neurite stimulatory media. Transmission electron microscopy revealed that neurites formed bundles, which were surrounded by larger cellular processes that were GFAP positive indicating a close association between growing axons and glial cells in this regeneration paradigm.

**Conclusions:**

We postulate that glial cells with increased *Gfap* expression support the elongation of new neurites from retinal ganglion cells possibly by providing a scaffold for outgrowth.

## Findings

### Introduction

Within the central nervous system, glial cells provide critical support for neurons. Due to the intertwined nature of glial and neuronal interactions and functions, when neurons are injured, as the retina ganglion cells (RGCs) are in glaucoma, glial cells also react and undergo morphological changes and alterations in gene expression [1-5].

After optic nerve injury astrocytes surrounding the optic nerve head become reactive and are intimately involved with formation of glial scar tissue in the optic nerve. The glial scar strongly inhibits axon regeneration from the RGCs and may contribute to further axon damage leading to RGC death and irreversible blindness [6-8]. Müller cells respond to optic nerve injury by increasing their expression of glutamine synthetase [9] and the growth factor ciliary neurotrophic factor [10]. One common feature of both astrocyte and Müller cell reactivity (and glial cell reactivity in general) is the increased expression of glial fibrillary acidic protein (GFAP) [9,11-17]. GFAP is a type III intermediate filament protein component of the cytoskeleton. Astrocytes constitutively express GFAP, while mature Müller cells normally do not express GFAP [1,3].

Beyond GFAP’s function as a cytoskeleton component, its role within glial cells is poorly defined, particularly with regard to its ability to influence neuron specific events like axon regeneration. In animals that are able to regenerate axons, like zebrafish, goldfish, and lizards, GFAP positive outgrowths do not inhibit normal axon formation in development or axon regeneration after injury [6,18-21]. In co-cultures of mammalian cortical neurons and astrocytes however, astrocytic production of GFAP appears to suppress neurite outgrowth, since knocking-out GFAP production in astrocytes significantly improves neurite outgrowth [22-25]. Similarly, studies with *Gfap* and *Vimentin* double knock-out mice (*Gfap*^-/-^*Vim*^-/-^) indicate that reactive glia suppress integration and neurite extension of transplanted retinal neurons in the mouse eye [26]. However, GFAP-null mice showed no improvement in axon regeneration compared to wild-type mice after dorsal hemisection of the spinal cord [27].

In general, increased GFAP expression in glia is viewed to be detrimental to axon regeneration from neurons. This view is being challenged with work that demonstrates that the context of GFAP expression in the activated glia may be more important than the absolute levels of GFAP expression. In a rat axotomy model, administration of hydrocortisone and autocarboxyilic acid (an anti-apoptotic agent) significantly increased axon regeneration and GFAP expression, while also increasing expression of beneficial glial molecules like glutamine synthetase [28]. In a rat optic nerve crush model where zymosan was intravitreally injected, macroglia were highly stimulated as evidenced by a strong increase in GFAP positive cells, and there was also a significant increase in the number of neurites from RGCs [29]. This effect was partially mediated by increased expression of apolipoprotein E by the macroglia. In a model where Müller glia were constitutively active and GFAP positive, there was no evidence of negative effect on retinal neurons in terms of function [16]. Recent work has shown that activated GFAP positive retinal macroglia from glaucomatous rat eyes enhance axon regeneration from RGCs stimulated by both membrane-bound and soluble factors [17]. This raises the important point that while GFAP expression may be an excellent marker for glial activation, the context of that activation in terms of the suite of other molecules expressed and the cell type being examined may be more important in determining whether the glial cells are supportive or detrimental to axon regeneration. This may be especially important within the retina because of the Müller glia population, which supports the entire neural architecture of the retina in a way that is distinct within the central nervous system.

We have reported that in a retinal explant model of RGC axon regeneration, treatment with a well-defined combination of molecules (EGF, FGF2, insulin, biotin, transferrin, putrescine, progesterone, and hydrocortisone) in the absence of serum produced a significant increase in both neurite outgrowth and *Gfap* mRNA abundance [30]. In this study our goal was to utilize the retinal explant model to directly examine the effect of GFAP on neurite outgrowth by titrating the amount of GFAP expression in the explants. Hydrocortisone increased *Gfap* promoter activity and GFAP protein levels in the explant system. The amount of GFAP expression in the system was further manipulated by using explants from retinas of transgenic mice expressing GFAP at levels varying from none up to 2 times normal. Knocking-out or reducing GFAP had no beneficial effects on neurite outgrowth from explants compared to those with normal endogenous GFAP levels. Over-expression of GFAP was beneficial to neurite outgrowth, but only under conditions that were overall stimulatory for this process. Examination of explant sections via transmission electron microscopy revealed that axon structures appeared to be bundled together into larger fibers and that these bundles were ensheathed by glial cellular processes. Overall these data indicate that in the retina GFAP is not detrimental to axon regeneration and in fact might be associated with support of new neurite outgrowth under certain conditions.

### Materials and methods

#### Animals

Animals were handled in accordance with the Association for Research in Vision and Ophthalmology Statement on the use of animals for research and approved by the University of Wisconsin Institutional Animal Care and Use Committee. Strains of mice used included CB6F1, FVBB6F1 (*Gfap*^*+/+*^), transgenic m*Gfap* promoter luciferase reporter strain (FVB/N-Tg(*Gfap-luc*)-Xen (Caliper Life Sciences, Hopkinton, MA)), *Gfap*-null mice (*Gfap*^*tm1Mes*^ that are congenic in either C57BL/6J or FVB/N backgrounds, or *Gfap*^*-/-*^ and *Gfap*^*+/-*^ in either the two backgrounds) [31], transgenic *mGfap*-wt over-expressing mice (FVB/N-Tg(170.2), Tg170.2). In each case, female mice in the FVB/N background were crossed with male C57BL/6 mice to generate *Pde6b*^*rd1/+*^ offspring for use in experiments.

Genotypes for mice were confirmed by PCR. For *mGfap-Luc* mice, genotyping was performed using the PCR protocol supplied by the manufacturer (forward primer 5^′^- TGGATTCTAAAACGGATTACCAGGG-3^′^ and reverse primer 5^′^- CCAAAACAACAACGGCGGC-3^′^, Caliper Life Sciences). For *Gfap*^*tm1Mes*^ mice, genotyping was performed using the PCR protocol supplied by the Jackson Laboratory (Bar Harbor, ME) (common forward primer 5^′^- GATGGAGCGGAGACGCATCACC-3^′^, wild type reverse primer 5^′^-TTGTCCCTCTCCACCTCCAGCC-3^′^, or mutant reverse primer 5^′^-GGAAGACAATAGCAGGCATGCTGG-3^′^). For Tg170.2 mice, genotyping was performed using forward *mGfap* 5^′^ promoter primer 5^′^- ACTGCACCCGGGGCTGACATCCTG-3^′^ and 5^′^ loxP site reverse primer 5^′^- GAGTTGGCTGTGCATGCATAACTTCGTATAAT-3^′^.

#### Retinal explant protocol

Retinal explants from postnatal day 7 (PN7) mice were harvested and embedded in collagen matrices as previously reported [30]. Eight explants were taken from each eye for a total of 16 explants from each mouse. 150 μL of the appropriate supplemented media was added on top of explants with 4 explants per individual mouse per media. Supplemented media included 10% FBS (BioWhittaker, Walkersville, MD), EN2 (10% N2 (Invitrogen, Carlsbad, CA) with 1μg/mL biotin (Invitrogen), 0.36 μg/mL hydrocortisone (HC) (Sigma-Aldrich, St. Louis, MO), 0.5 μg/mL FGF2 (Invitrogen), and 1μg/mL EGF (Invitrogen)) or EN2 without hydrocortisone (EN2 w/o HC) all in DMEM with 1% PenStrep (BioWhittaker). Explants were cultured for 7 days at 37°C with 5% CO_2_. Media was replaced every other day. The number of neurite outgrowths from each explant was counted every 24 hours under phase contrast optics using a Leitz DM IL microscope (Microsystems, Inc., Buffalo Grove, IL) and the mean (± SEM) number of neurites was determined for each treatment group. After 7 days in culture, explants were washed for 10 minutes in phosphate buffer saline (PBS) at room temperature. Explants were then either fixed for transmission electron microscopy or frozen at -80°C for luciferase assays or ELISA.

#### Gfap Luciferase reporter assays

Luciferase assays were performed using the Promega Luciferase Assay System (Promega, Madison, WI) with some modifications for extracts from mouse retinal explants. Explants used for luciferase assays were placed in 150 μL of 1X reporter lysis buffer (Promega, Madison, WI) and put through 3 freeze/thaw cycles (-80°C/22°C) [32]. The total contents of each well were transferred to microcentrifuge tubes and centrifuged at 13,200 x *g* at room temperature for 2 minutes. 100 μL of luciferase assay reagent was mixed with 20 μL of explant lysate in a luminometer tube and the luminescent signal was measured using a Turner TD-20e manual luminometer (Turner BioSystems, Sunnyvale, CA). Lysate samples were assayed in triplicate.

#### ELISA for GFAP quantification

Retinal samples from *Gfap*^*-/-*^, *Gfap*^*+/-*^, *Gfap*^*+/+*^, and Tg170.2 mice were prepared by dissecting out the retinas from each mouse and pooling the two retinas together, freezing the retinas overnight at -80°C, thawing the retinal sample, and homogenizing each pooled sample in 0.2 mL lysis buffer (2% sodium dodecyl sulfate, 50 mM Tris–HCl, 5 mM EDTA, pH 7.4, 1 mM phenylmethylsulfonyl fluoride and Complete Proteinase Inhibitor Cocktail (Sigma)). The retinal lysates were boiled for 15 minutes then diluted 1:10 in 0.5% Triton-X 100 and 1% BSA in PBS. Diluted retinal lysate and undiluted explant lysate were quantified for GFAP content using a sandwich ELISA as previously described [33] with the SMI-26 anti-GFAP monoclonal antibody cocktail (Covance Research Products, Emeryville, CA) as the capture antibody and a polyclonal rabbit anti-cow GFAP (DAKO, Carpinteria, CA) as the detection antibody. ELISAs were read with a Tecan Safire^2^ microplate reader (Tecan US, Inc, Durham, NC). Retinal lysate samples were assayed in triplicate then normalized to the total sample protein concentration as determined by bicinchoninic acid protein assay (Thermo Scientific Pierce, Rockford, IL) performed according to the manufacturer’s instructions.

After thawing, explants for each mouse in a given treatment condition (four explants per mouse per treatment group) were pooled in 400 μL lysis buffer and homogenized. The lysate was boiled for 15 minutes. Undiluted explant lysate was quantified for GFAP content using the sandwich ELISA described above. Explant lysate samples were assayed in triplicate and the amount of GFAP was analyzed on a per explant basis as was the neurite outgrowth since explant size did not vary significantly as demonstrated previously [30].

#### Transmission electron microscopy

Explants used for standard TEM were fixed with 200 μL/well of 2.5% glutaraldehyde, 2% paraformaldehyde in 0.1 M phosphate buffer (PB) for 48 hours at 4°C. Fixed explants were post-fixed in 1% osmium tetroxide in PB, dehydrated in ethanol, and embedded in Epon epoxy. Sections (60 nm) were cut, stained with 50% ethanoic uranyl acetate and Reynold’s lead citrate and viewed using a Philips CM120 transmission electron microscope (FEI Company, Hillsboro, OR). Identification of structures on the ultrastructural level was based on criteria established by Hogan et al [34].

Some explants were immunolabeled for GFAP prior to embedding using the Aurion ImmunoGold pre-embedding labeling method. The explants were first fixed in 200 μL/well of 0.1% glutaraldehyde, 2% paraformaldehyde in PB overnight at 4°C. Fixed explants were incubated in 0.1% NaBH_4_ in PB for 10 minutes then washed 4 times in PB for 5 minutes. Explants were permeabilized with 0.1% Triton-X-100 in PBS for 30 minutes then washed 3 times in PBS for 10 minutes. The explants were blocked in Aurion blocking solution (Aurion, Wageningen, Netherlands) for 1 hour then washed 3 times in incubation buffer (IB, 0.1% Aurion BSA-c (Aurion) in PBS) for 10 minutes. Rabbit monoclonal anti-cow GFAP (DAKO) was diluted to 5 μg/mL in IB and explants were incubated in the primary antibody overnight at 4°C. Explants were washed 6 times in IB for 10 minutes, then incubated with ultra small gold conjugated goat anti-rabbit IgG (1:100 in IB, Aurion) overnight at 4°C. The explants were washed 6 times in IB for 10 minutes, washed with PBS 6 times for 5 minutes, post-fixed in 2% gluteraldehyde in PB for 30 minutes, washed in PB 2 times for 5 minutes, and finally washed with distilled water 3 times for 5 minutes. The explant samples were incubated in silver enhancement mixture (Aurion) for 40 minutes then washed 3 times in distilled water for 10 minutes. Labeled explants were then prepared for TEM sectioning and visualization as described above for standard TEM.

#### Statistics

A minimum of 24 explants was evaluated for each treatment per experiment, representing 8 retinal explants from 6 individual mice in all treatment groups. Experiments were repeated 2-3 times. Means are reported with the standard error of the mean (SEM). Statistical significance between treatments was determined using Prism 5 software (GraphPad, La Jolla, CA) for two-way ANOVA, one-way ANOVA with Newman–Keuls ad-hoc post-test for individual comparisons, or Student’s t-tests, P < 0.05.

### Results

#### Effect of HC on GFAP in retinal explants

**Figure 1:**
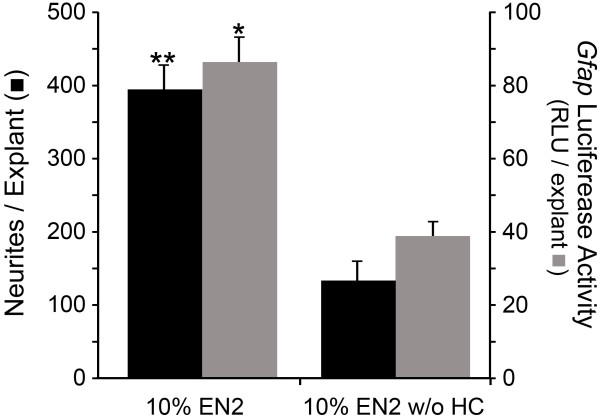
**HC increased *Gfap *promoter activity and neurite outgrowth from retinal explants.** Histograph of the mean number of neurites per explant (± SEM, black bar) and the *Gfap* promoter driven luciferase activity as measured by the mean relative fluorescent units (RLU) per explant (± SEM, grey bar) in transgenic PN7 retinal explants at culture day 7 treated with either 10% EN2 or 10% EN2 without HC (EN2 w/o HC). EN2 treated explants had significantly more *Gfap* promoter activation (*P<0.001) and neurites (**P=0.031) than EN2 w/o HC treated explants. Significance was determined by Student’s *t*-test, *p*<0.05.

We have shown previously that *Gfap* mRNA expression was increased in PN7 retinal explants treated with EN2 (containing HC) compared to EN2 without HC (EN2 w/o HC) at both 4 and 7 days in culture [30]. Explants from transgenic mice expressing firefly luciferase driven by a 12.2 kb mouse *Gfap* promoter had significantly more luciferase activity after 7 days in culture when grown in EN2 compared to EN2 w/o HC (P<0.001, Figure 1), as well as significantly more neurites than transgenic explants in EN2 w/o HC (P=0.031, Figure 1). These data suggest that increased accumulation of *Gfap* mRNA was a consequence of HC-mediated transcriptional activation of the *Gfap* promoter. Immunostained frozen sections of explants at both 4 and 7 days in culture showed that GFAP labeling was increased principally in Müller glial cells in EN2-treated explants, relative to EN2 w/o HC-treated samples (data not shown).

#### Titration of GFAP in explants

**Figure 2:**
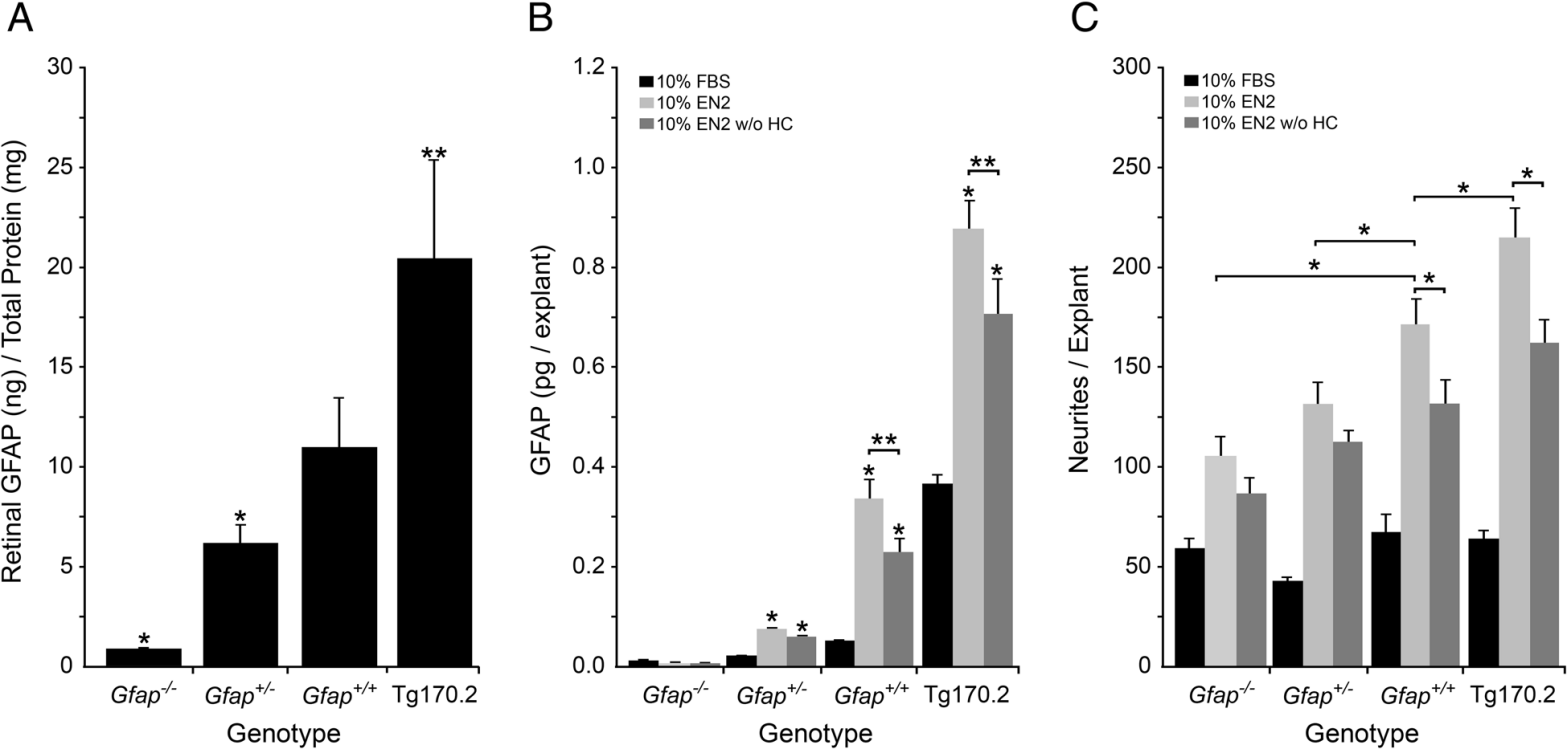
**GFAP levels are positively associated with neurite outgrowth under growth stimulatory conditions.** Histographs showing from different mouse genotypes at PN7 (**A**) the amount of retinal GFAP (mean±SEM), (**B**) GFAP levels (mean±SEM) in PN7 retinal explants cultured 7 days with either 10% FBS, 10% EN2, or 10% EN2 w/o HC, and (**C**) neurites per explant (mean±SEM) in PN7 retinal explants cultured for 7 days with 10% FBS, 10% EN2, or 10% EN2-HC. (**A**) The amount of GFAP in different mouse genotypes shows titration of GFAP from background levels (*Gfap*^*-/-*^, *P<0.001 versus *Gfap*^+/+^ retinas), to half the amount of GFAP (*Gfap*^*+/-*^, *P=0.048 versus *Gfap*^+/+^ retinas), to double the amount of GFAP (Tg170.2, **P=0.041 versus *Gfap*^+/+^ retinas). (**B**) In retinal explants cultured for 7 days the same relationship between GFAP levels and genotype was maintained, regardless of culture conditions (*P<0.05), although overall GFAP levels were significantly higher in explants cultured in EN2 versus EN2 w/o HC treated explants (**P<0.03). (**C**) None of the FBS treated explants had significant neurite outgrowth, indicating that increased GFAP alone does not increase neurite outgrowth. EN2 and EN2 w/o HC treated explants had increased neurites in all strains compared to FBS. In *Gfap*^+/+^ and Tg170.2 explants, there were significantly more neurites in EN2 treated explants than EN2 w/o HC treated explants. In *Gfap*^*-/-*^ and *Gfap*^+/-^ explants, this treatment dependent effect was ablated, indicating that one important contribution of HC to increased neurite outgrowth is the increase in GFAP. Bracketed comparisons are significantly different (P<0.05).

To test directly whether *Gfap* expression was beneficial or detrimental to neurite outgrowth, we titrated the amount of GFAP in the explants by using transgenic mice expressing different levels of GFAP, from *Gfap* knock-out mice (*Gfap*^-/-^) to *Gfap* over-expressing mice (Tg170.2). Initial levels of GFAP in the PN7 mice were determined in the different lines by measuring the amount of GFAP protein from PN7 retinas (*Gfap*^-/-^, *Gfap*^+/-^, *Gfap*^+/+^, and Tg170.2 mice) by ELISA (Figure 2A). ELISA results confirmed that *Gfap*^-/-^ mice had no detectable GFAP in their retinas above background (P<0.001 compared to *Gfap*^+/+^ mice) and that *Gfap*^+/-^ mice had half the amount of GFAP in their retinas as *Gfap*^+/+^ mice (P=0.048). The Tg170.2 mice had twice the amount of GFAP in their retinas compared to *Gfap*^+/+^ mice (P=0.041).

After 7 days in culture, the amount of GFAP (Figure 2B) and the number of neurites (Figure 2C) was determined for *Gfap*^-/-^, *Gfap*^+/-^, *Gfap*^+/+^, and Tg170.2 retinal explants cultured in either 10% FBS, 10% EN2, or 10% EN2 w/o HC. The previously reported increase in *Gfap* mRNA transcripts in wild-type EN2 treated retinal explants compared to EN2 w/o HC treated explants [30] corresponded to a significant increase in GFAP in the EN2 treated *Gfap*^+/+^ explants compared to EN2 w/o HC treated explants (P=0.0252).

In explants cultured in poor growth promoting media (10% FBS), there was no significant association between neurite outgrowth and GFAP levels, while in explants cultured in growth permissive media (10% EN2 or 10% EN2 w/o HC), however, there was a clear association between the level of GFAP protein and neurite outgrowth in the retinal explants (Figure 2C, two-way ANOVA row (treatment) and column (genotype) factors both significant, P<0.0001). GFAP levels were significantly higher in explants grown in EN2 of all genotypes, relative to those cultured in EN2 w/o HC (Figure 2B, one-way ANOVA, P<0.05). Neurite outgrowth was also greater in the EN2-treated explants (Figure 2C, P<0.05), but only in genotypes with the capacity to express high levels of *Gfap* (wild type and Tg170.2 over-expressing mice). The *Gfap*^-/-^ and *Gfap*^+/-^ explants showed no significant difference in neurite outgrowth between EN2 and EN2 w/o HC conditions (Figure 2C, one-way ANOVA, P>0.05). Overall, explants with the greatest levels of GFAP expression showed a concomitantly increased capacity for neurite outgrowth.

#### Examination of the ultrastructure of retinal explants

**Figure 3:**
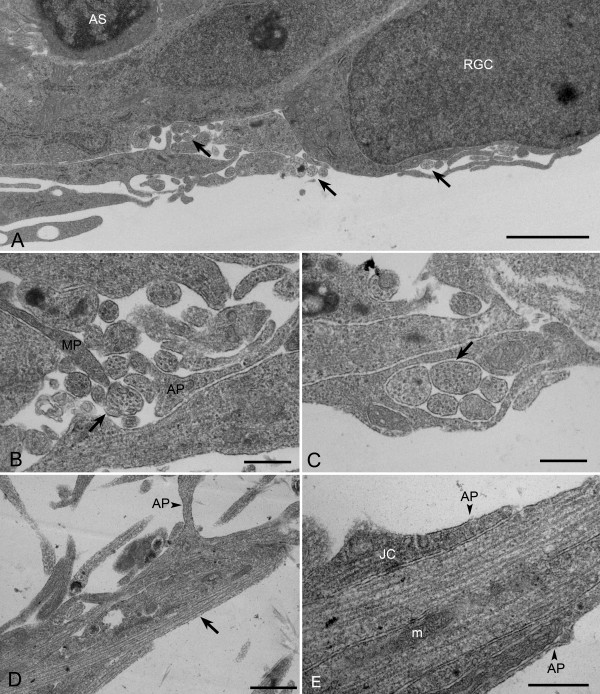
**Neurites grow in bundles and are surrounded by cellular processes.** (**AE**) Electron micrographs of retinal explants grown in EN2 for 4 days. (**A**) Low magnification image of the edge of the explant in cross-section shows the close association of retinal ganglion cell (RGC) and putative astrocyte (AS) cell bodies as well as several neurite bundles (arrows) surrounded by cellular processes. (**BC**) Higher magnification images of areas both near (**B**) and distant (**C**) to the explant in cross-section show neurite bundles (arrows) surrounded by cellular processes, some of which appear to be putative Müller cell processes (MP) and putative astrocyte processes (AP) (**B**). (**DE**) Low magnification (**D**) and high magnification of a distal process from the explant in longitudinal section shows a microtubule rich neurite axonal process (arrow) in contact with astrocyte processes (AP). A junction complex can be seen between the neurite process (JC) and putative astrocyte process (AP) in (**E**). Identification of putative glial processes were based on criteria established by Hogan et al [34]. Scale bars equal 2 μm (**A**), 500 nm (**BC**, **E**), and 1 μm (D), m=mitochondria.

In samples of wholemount explants that were immunolabeled for beta-III tubulin and GFAP, it appeared that neurites were closely associating with glial processes (data not shown). We were unable to achieve sufficient resolution with our immunofluorescent samples, due to the thickness of the collagen the explants were embedded in, to determine whether glial processes may have acted as scaffolds to support neurite outgrowths. We therefore pursued TEM sectioning of explants to determine if axonal structures were associating with other cellular processes. Cross-sections of explants revealed axonal structures rich in microtubules (Figure 3A-C). Axons were generally found in clusters. Groups of axons were intimately associated with cellular processes, which appeared to surround developing axon bundles. Longitudinal sections of neurites examined at a distance from the main body of the explants showed axonal structures rich in microtubules (Figure 3D-E). These axonal structures were found to have separate cellular processes running parallel to them. In some places, tight junction structures appeared where the axons and cell processes made contact. Based on studies conducted by Hogan et al [34], axons were contacted by processes from both astrocytes (with light staining cytoplasm and distinct electron dense nuclear morphology) and Müller cell end feet (darker staining granular cytoplasmic appearance).

**Figure 4:**
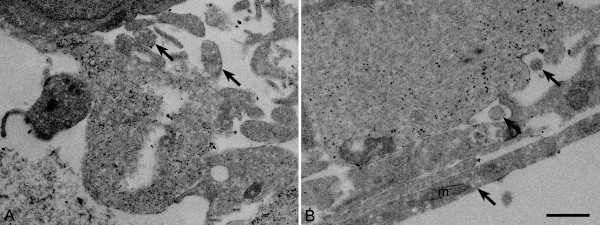
**Processes surrounding neurites are GFAP positive.** (**A**-**B**) Electron micrographs of cross-sections of explants grown in EN2 after 4 days in culture that have been Immunogold labeled for GFAP. In these images GFAP positive immunolabeling is visible as the electron-rich black dots in the electron micrographs. Axons (arrows) do not retain clear microtubule organization in samples processed for immunolabeling, but were identified on the basis of structure and size. Likely axonal processes are surrounded by GFAP positive glial processes. Scale bar equals 1 μm, m=mitochondria.

The identity of the non-axonal cellular processes interacting with the axons could not be determined by ultrastructure analysis alone. Immunogold labeling of GFAP was used to determine if the non-axonal cell processes were emanating from glia. Gold-labeled particles were found predominantly in cells in the RGC layer of explant cross-sections corresponding with the astrocytes and Müller cell endfeet (Figure 4). Gold-labeled particles were found in the same processes ensheathing the putative axonal bundles (Figure 4, note that microtubules were not visible since Immunogold labeled samples cannot be stained for visualization of electron dense structures).

### Discussion

Increased expression of GFAP is an important marker of glial activation after injury to the CNS [35]; however, what this increase means for the survival of surrounding neurons and the potential for axon regeneration from these neurons is unclear. While increased glial reactivity, as marked by increased GFAP, has been largely viewed as an negative outcome for neurons, there is evidence that this may not be the case and that glial reactivity and GFAP expression changes must be viewed in the context they occur. GFAP could be positively influencing the ability of neurons to regenerate by altering the localization of proteins that can interact with neurons at the glial cell membrane; for example, GFAP has been shown to aid in membrane retention of the glutamate transport GLAST in astrocytes which protected surrounding neurons from glutamate excitoxicity [36]. There may also be a role for GFAP in controlling the expression of other secreted molecules, like the growth factor GDNF or the extracellular matrix protein laminin, that alter axon regeneration by neurons [22,37,38]. Lastly, GFAP positive glial processes could be acting as scaffolds for new neurites by providing cell-cell interactions that enhance RGC neurite outgrowth and pathfinding [17,39].

We previously showed that HC, as a part of a neurite stimulatory media, increased *Gfap* mRNA transcripts and GFAP positive labeling of astrocytes and Müller cells in the explants. This was associated with an increase in the number of neurite outgrowths from explants. Here we show that HC leads to an increase in *Gfap* expression by interacting with the *Gfap* promoter and that the increase in *Gfap* transcripts also leads to increased GFAP. To directly test whether GFAP was beneficial or detrimental to neurite outgrowth in the explant culture paradigm, we titrated the expression of *Gfap* using transgenic mice expressing a range of GFAP levels. Knocking out or reducing the amount of GFAP did not increase the amount of neurites compared to wild-type explants, regardless of treatment, and in fact had the opposite effect (at least when comparing complete nulls vs. wild-type). These results contradict findings using co-culture paradigms of dissociated astrocytes and cortical neurons [22-24], and may reflect more complex cell interactions that are retained using the explant culture system. Alternatively, different populations of neurons may interact differently to GFAP-expressing macroglia. In the case of retinal ganglion cells, which produce long projection axons, extended glial support scaffolds may be a critical component for successful neurite outgrowth.

Increasing the amount of GFAP above wild-type levels resulted in the most neurite outgrowth, but only in a stimulatory media, like EN2. Increased GFAP concentration alone was insufficient to stimulate increased neurite outgrowth in a non-stimulatory media, like FBS. Treating the explants with EN2 resulted in the most neurite outgrowth regardless of genotype, consistent with previous findings. In total these data indicated that, under some conditions, GFAP is not detrimental to new neurite outgrowth and that over-expression of GFAP may actually support new neurite outgrowth.

The mechanism of neurite outgrowth enhancement and stabilization by *Gfap* over-expression is unclear. The Tg170.2 mice by design over-express mouse *Gfap*, but whether they display other features of activated astrocytes is not yet clear. One possible mechanism may be more robust glial interactions with growing neurites, which could be augmented by GFAP-cytoskeletal interactions that support more or stronger glial processes. Several reports indicate that growing axons track along GFAP-positive glial processes during development, although these are largely observed in invertebrate and anamniontic animal development [21,39,40]. We were able to detect this close association in explants at the ultrastructural level. Neurites were often detected in bundles that were ensheathed by glial cellular processes.

The idea that glial cells could act as scaffolds for new axons has been postulated as a possible solution for axon regeneration within the spinal cord [27,41,42]; however, this has not been extensively studied within the retina, which is surprising considering how closely astrocytes are associated with the formation of the glial scar [3,6]. Recent work indicates that retinal glia expressing GFAP [17] or GFAP and nestin [43] may provide structural support to RGCs. Our data show that increased GFAP can play an important part in increased neurite outgrowth, possibly by augmenting glial interactions with regenerating axons and potentially serving as a scaffold for new axon outgrowth. In this model, GFAP is predicted to be part of a suite of molecules whose expression profiles are altered after glial activation to create a regenerative environment. We have shown previously that proteins like GLUL and CNTF appear to be part of this suite [30]; however, many additional candidate proteins remain to be studied including additional growth factors, neurotrophins, and other components of the cytoskeleton like nestin and vimentin. Future work with the *Gfap* over-expressing mice will be aimed at determining if there is alteration in the normal expression of growth factors, neurotrophins, and other components of the cytoskeleton in the glia that may indicate. This will determine if these cells are in a perpetually activated phase that is beneficial to neurite outgrowth and whether this is a phenomenon that is confined to the unique glial cell population in the retina. In conclusion, our work demonstrates that the context in which GFAP is increased is important and that the nature of the neural injury, the duration of the injury stimulus, the time point examined after injury, and the type of neuroprotective strategy taken will influence whether the glial response is beneficial or detrimental.

## Competing interests

The authors declare that they have no competing interests.

## Authors’ contributions

KT carried out the dissections to generate retinal explants, performed the neurite outgrowth experiments, the *Gfap* luciferase assays, the GFAP ELISAs, prepared the samples for TEM, performed the statistical analysis, and drafted the manuscript. TH and AM derived and provided the transgenic *Gfap* mice, provided the genotyping and GFAP ELISA protocols, participated in the design and analysis of the experiments, and helped to draft the manuscript. RN conceived of the study, and participated in its design and coordination and helped to draft the manuscript. All authors read and approved the final manuscript.
